# Effect of shade and precipitation on germination and seedling establishment of dominant plant species in an Andean arid region, the Bolivian Prepuna

**DOI:** 10.1371/journal.pone.0248619

**Published:** 2021-03-31

**Authors:** Natalio Roque Marca, Ramiro Pablo López, Kazuya Naoki

**Affiliations:** 1 Carrera de Biología, Universidad Mayor de San Andrés, La Paz, Bolivia; 2 Instituto Experimental de Biología “Dr. Luis Adam Briançon", Universidad Mayor, Real y Pontificia de San Francisco Xavier de Chuquisaca, Sucre, Bolivia; 3 Instituto de Ecología, Carrera de Biología, Universidad Mayor de San Andrés, La Paz, Bolivia; 4 Herbario Nacional de Bolivia, La Paz, Bolivia; Helmholtz Centre for Environmental Research - UFZ, GERMANY

## Abstract

Germination and seedling establishment are two critical processes in the life cycle of plants. Seeds and seedlings must pass through a series of abiotic and biotic filters in order to recruit as members of their communities. These processes are part of the regeneration niche of the species. In arid regions, the regeneration niche is frequently associated to facilitation by shade. Facilitation is a positive interaction between plants, in which one of them acts as a benefactor (the nurse) of the other (the beneficiary). The result of this interaction can be reflected in the increased growth, survival, and/or reproduction of the beneficiary plant. In this study, we determined experimentally the effect of shade and irrigation on the germination and early survival of dominant species of a semi-arid Andean region, the Bolivian Prepuna. An experiment with *Acacia feddeana*, *Prosopis ferox*, *Cercidium andicola* (woody species), *Parodia maassii*, and *Oreocereus celsianus* (cactus species) was carried out at an experimental garden in La Paz, Bolivia, with a bifactorial design, considering shaded and unshaded pots, subjected to two irrigation treatments (≈50 and 80 mm of rainfall during the whole study period). Microenvironmental conditions did not affect the seed germination of the woody species. However, they showed differences in seedling survival: *A*. *feddeana* survived better under shade, whereas *P*. *ferox* and *C*. *andicola* survived better without shade. *Cercidium* andicola, compared to *P*. *ferox*, was more affected by shade and low irrigation. Although germination success of cacti was low, both species germinated better under shade and with high irrigation. These results showed differences in the regeneration niche of dominant species of the Prepuna which may favor their coexistence and which may be characteristic of other dry Andean regions.

## Introduction

Plant regeneration comprises a life cycle from seeds to seedlings and adult plants [1, 2]. Within this cycle, two critical processes are germination and seedling establishment, as they determine population dynamics and persistence. Seeds and seedlings must pass through a series of abiotic and biotic filters in order to recruit as members of the community [3, 4]. However, the way plants face these filters may offer the possibility of interspecific coexistence by allowing species to differentiate along niche axes by possessing a different regeneration niche [5, 6].

One important aspect in the regeneration niche of desert and semi-desert species is the specific microhabitat conditions required by woody and herbaceous species for their successful germination and early establishment [7, 8]. In many species, shaded conditions are necessary for seedlings to germinate and/or survive, especially for cacti [9, 10]. Shade is mostly provided by nurse species, which are larger species that modify the microenvironment beneath them, and sometimes by inanimate objects, such as rocks or dead plant material [11–13]. This positive effect of nurses is considered one of the main mechanisms that underlies facilitative relationships between plants [14], and is one of the most important plant—plant interactions in habitats with extreme environmental conditions [15, 16]. The facilitating effect of nurse plants appears to be more important during germination and establishment of beneficiary species [10, 17], and the positive effect frequently disappears or becomes competitive in later phases of the beneficiary’s life cycle [18–20].

Interspecific facilitation mediated by shade has arisen enormous interest due to its influence on the functioning of ecosystems [15, 21], especially in those with abiotic stress, such as deserts [22, 23]. For example, high radiation and low precipitation during the summer or dry season in arid regions is dangerous for the photosynthetic capacity of the seedlings (photodamage) and exposes them to water stress [24]. The shade provided by the canopy of trees or shrubs benefits plants [25, 26] by reducing the temperature, and favorably alter soil water availability [21, 27, 28] by decreasing evaporation rates [9, 29]. In addition, shade has effects on the photosynthesis and morphological plasticity of plants [30, 31].

It is common to observe in arid and semi-arid ecosystems higher plant recruitment under tree or shrub patches compared with open spaces [32, 33]. However, the effect of shade can be variable depending on the amount of water present in the system [10]. If there is enough water, seedlings may grow in one or another environment (shade/open), or survival/growth may even be improved in open spaces, as this would reduce competition with the nurse plant. This interaction between shade and water is of vital importance for understanding the mechanisms of facilitation and for species coexistence.

The so-called “drylands” include all those ecosystems from desert to semi-arid environments, and represent 41% of the planet’s surface [34, 35]. Arid regions are generally characterized by low and variable rainfall, extreme environmental temperatures and high potential evapotranspiration [36, 37]. South America has different dryland types. Among these, the Atacama Desert stands out [38–40]. This desert is dominated by El Niño events [41–43]. On the other hand, there are deserts with summer rains, such as the Monte desert and the Prepuna. The Atacama Desert and the Monte Desert are relatively well-studied; however, the more septentrional regions, and namely the Prepuna, are poorly known [44–46]. Moreover, the Prepuna has a relatively small area and is patchily distributed along the more arid parts of Andean valleys, and it is located in a region predicted to become more arid [47, 48], making it an endangered biogeographical region.

The study of dominant plant species is important in order to understand how a community is organized, especially in arid environments where a few species represent the bulk of the biomass and determine community dynamics. Understanding how dominant species get established in a given community is fundamental to get insight into their regeneration niche, and shade plays a role in this sense. Besides its positive effect on a plant’s water economy, shade may have contrasting effects on germination and survival [49], but little is known for xeric Andean environments, and summer rain deserts in general. In this paper we aim at understanding how water and shade availability can interact and affect seed germination and early establishment of five dominant species of the Bolivian Prepuna, three woody (*Acacia feddeana*, *Cercidium andicola*, *Prosopis ferox*) and two succulent species (*Oreocereus celsianus*, *Parodia maassii*). The three woody species constitute the largest biomass of most of the communities in this region; thus, studying their behavior in their early life stages seems essential in order to understand the functioning of dry environments in the Andes. We predict that shade may improve seed germination and seedling survival of the species studied. Due to the interaction between water and shade availability, individuals under shade with low precipitation may respond in a similar way as those growing without shade but high precipitation, which would suggest that shade buffers the negative impacts of dry years on seedling establishment of dominant species in the Prepuna.

## Materials and methods

### Seed origin

The seeds of shrub/small trees and cactus species used for this study are considered resistant to drought and cold [50]. They were obtained from three localities in the northern-most part of the Prepuna biogeographical region, which is found between 20 and 22°S in Bolivia. The Prepuna also extends between 23 and 27°S in Argentina [51]. In the Bolivian Prepuna, the climate is semi-arid with a marked seasonality. Annual rainfall is between 220 and 350 mm [52], and the average annual temperature ranges from 12 to 18°C depending on elevation. During winter, the temperature may reach several degrees below zero in the early morning, and during summer the temperature can go up to almost 40°C [28]. Vegetation is composed of patches of shrubs located within a predominantly open habitat of bare soils, stones, and small herbs, where woody cover occupies ca. 20–40%. The shrub patches cast shade, and several woody and herb species grow in those more mesic conditions [28, 53].

The seeds of all five species were obtained at three localities (20°45’4” S, 65°37’50” W, 3119 m a.s.l.; 21°21’28” S, 65°3’13” W, 3023 m a.s.l.; and 20°9’33” S, 65°18’21” W, 3338 m a.s.l.). Mean temperature is 14.5°C, and annual rainfall is 310 mm, 90% of which falls between the end of the spring and the end of summer, at the nearest weather station in Tupiza, at ca. 90 km from the study localities with a similar altitude of 2620 m a.s.l. [54]. In 2010, at each locality at least 10 individuals of each species were sampled, and as many fruits (pods for the trees and berries for the cacti) as possible were collected (around 10–20 fruits per individual; for *Cercidium andicola* only 5–10 fruits per individual were taken because of low fruit production). Seeds were transported to the Universidad Mayor de San Andrés, in the city of La Paz (3400 m a.s.l.), where the experiment took place, and were stored in a dry, cool place. In 2011 the same protocol was repeated for the woody species in order to obtain more seeds.

### Study species

All five species are important components of the Bolivian Prepuna in terms of cover and/or density. Three of them are also common in the Argentinean Prepuna (*Prosopis ferox*, *Cercidium andicola*, *Parodia maassii*; *Oreocereus celsianus* occurs also there near the Bolivian frontier). *Acacia feddeana* Harms (Mimosoideae), locally known as palqui, is endemic to the Bolivian Prepuna. This species was described as a shrub or small deciduous tree 2 to 4 meters high. *Prosopis ferox* Griseb. (Mimosoideae), known as churqui in the study area, is a tree up to 6 m high, having one main trunk or several basal branches (shrubby habit). For germination, seeds must undergo mechanical scarification or the digestive tract of some ruminants [50, 55]. *Cercidium andicola* Griseb. (Caesalpinioideae), known as ckatawi or sinqi [50], is a small, deciduous tree up to 4 m high. *Oreocereus celsianus* (Lem. ex Salm-Dyck) Riccob. (Cactaceae), known as puli-puli or wirka in Potosí, Bolivia, is a succulent plant up to 4–5 m high, branched at the base, and sometimes at the top; it has cylindrical, erect, and hairy stems with shiny, golden thorns white hair. *Parodia maassii* (Heese) A. Berger (Cactaceae) is a globular cactus that has an intense green spherical or oblong stem with the apical part densely covered with white woolly hairs. It reaches ≈25 cm in height.

### Experiment

The experiment was carried out in the wet season at the botanical garden of the Universidad Mayor de San Andrés, in La Paz city (3400 m a.s.l.), where mean temperature is 13.6°C, and annual precipitation is 428 mm [54]. The fruits of each species from the three localities and both years were mixed to avoid the among-population variation affecting the experiment. The fruits of all five species were dried at room temperature. The seeds of *C*. *andicola* and *A*. *feddeana* were naturally released from the dehiscent fruit, and the seeds of *Prosopis ferox*, *Oreocereus celsianus*, and *Parodia maassii* were extracted mechanically because their fruits are fleshy (cacti) or are indehiscent (*P*. *ferox*) and do not open naturally. *Prosopis ferox* seeds were scarified with 98% sulfuric acid for 8 minutes and stored in a glass jar [55, 56].

The experiments of seed germination and seedling survival were conducted in a 3 m x 1.5 m area, where 200 cylindrical plastic pots were placed. The pots had a diameter of 8 cm and a height of 12 cm and were filled with nutrient-poor sandy soil, similar to one found in the Prepuna [57]. The 40 plastic pots for each species were placed together as 10 rows X 4 columns. We placed 20, 15, and 6 seeds of *P*. *ferox*, *A*. *feddeana*, and *C*. *andicola*, respectively, and 30 seeds of *O*. *celsianus* and *P*. *maassii*, at the depth of ca. 1 cm. The difference in the number of seeds used for the study depended on their availability in the field. The pots were covered with one big piece of glass located at 10 cm above them; hence, they were protected from birds and rains that may have altered the irrigation treatments.

A bifactorial experiment was conducted for each species in a randomized block design, in which each species was kept separated from one another, and each of the combinations of shade treatment and irrigation appeared once in each of the 10 rows. The two factors considered were shading levels (unshaded and artificially shaded) and precipitation levels (irrigation treatment for 50 and 80 mm), and there were 10 replicates per combination of each factor level, making a total of 40 experimental units per species. Therefore, 20 pots (unshaded) were exposed to daily radiation (simulating open places in the ecosystem), and the other 20 pots were covered with sheer nets that provided ≈70% artificial shade, simulating the shade generated by larger plants.

The nets help reproduce the positive effects of the nurse canopies without incorporating the negative effects of underground competition [58]. We prepared four pots (two shaded/two unshaded) for measurements of abiotic variables. Soil temperature was measured with a Kessler liquid-in-glass thermometer 0/100°C, and humidity was recorded with a KELWAY (0–100%) soil humidity recorder. Soil temperature and humidity were measured at 3 cm depth below the surface. Radiation was measured with a digital Mavolux luxometer (which measures up to 1000 lux) at the soil surface. All recordings were conducted in two pots per shading treatment at 10 times every two days during 20 days between 12:00 and 14:00, and the mean and standard deviation (SD) were calculated. Before the measurements, the same amounts of water as those in the pots containing seeds were added. Unshaded pots received solar radiation of 741.7 ± 176.7 Cd/m^2^, the soil temperature was 35.8 ± 3.6°C, and the soil moisture was 6.7 ± 1.2%. In contrast, shaded pots received less solar radiation of 472.6 ± 55.8 Cd/m^2^, the soil temperature was lower at 25.0 ± 1.6°C, and the soil moisture was higher at 15.2 ± 2.2%. Irrigation treatment level (≈50 and 80 mm) were chosen according to germination thresholds determined in previous studies [52, 59]. To calculate how much water should be added to each pot to achieve corresponding precipitation level (50 and 80 mm), we used the formula: the volume of water (ml) = the superior area of pots (4 cm * 4 cm * 3.14156) * the height (5 or 8 cm), which resulted 251 ml for 50 mm precipitation and 402 ml for 80 mm precipitation. On the first day, 88 ml of water (equivalent to 17.5 mm of precipitation) was added to each pot, and in the following days, we supplied 20–30 ml of water (equivalent to 4–6 mm of rain) every 2–3 days to keep the soil surface wet, until the total volume of 251 or 402 ml per pot was reached. We used a vial with small holes at the top so as to pour water gently over the entire soil surface. The irrigation concluded between the 36th and 45th day, when the equivalent of 50 and 80 mm of precipitation was attained.

Seed germination was recorded when the cotyledons appeared on the soil surface, and then each seedling was marked and followed during 30 days to check its survival in the same pot. To determine whether the seedlings were still alive, we recorded the withering state of each seedling [60]. The seeds germinated between the 6th and 30th day. Thus, the observation of seed germination and seedling survival lasted up to 60 days.

### Statistical analysis

#### Seed germination

The germination capacity (GC) was determined, using the following equation:

$$GC\left(\%\right)=\left(\frac{\sum E_{i}}{N}\right)\times 100$$

where *N* is the total number of seeds sown and *E*_*i*_ is the number of seeds that germinated each day [61, 62].

To analyze the effect of irrigation and shading on germination capacity, we applied generalized linear models (GLMs) with a binomial distribution and logit link function with the statistical program R version 3.6.0 [63]. When the over or under-dispersion was found according to the qq-plot and the dispersion parameter, we used beta-binomial distribution, which was implemented in the “aod” package [64]. In the global model of each species, the numbers of germinated and ungerminated seeds were treated as the dependent variables, and the irrigation (50 vs 80 mm), the shading (unshaded vs artificial shaded), and the interaction between these as the independent variables. For each species, reduced models were generated with all possible combinations of independent variables, and these models were sorted according to Akaike information criterion with a correction for small sample sizes (AICc) [65], using the “dredge” function of the “MuMIn” package [66]. In addition, the best model was compared with the null model by the likelihood-ratio test for each species.

#### Seedling survival

Seedling survival was analyzed using the Kaplan-Meier method (log Rank test; IBM SPSS Statistics version 19) and Cox proportional hazards model [67]. The Kaplan-Meier method was used to compare the survival curves under different microhabitat conditions. The analysis of survival curves was performed for the first 15 days to standardize the comparison among species because the majority of mortality occurred during this period for *P*. *ferox*, *C*. *andicola*, and the unshaded and low irrigation treatment of *A*. *feddeana*. We did not analyze the seedling survival of *O*. *celsianus* and *P*. *maassii* as there were few or no germinated seeds under some microhabitat conditions to follow their survival. Cox model was used to model the seedling survival time as a linear combination of irrigation, shading levels and the interaction between irrigation and shading levels. The following model was used:

$$h\left(t\right)=\left[h_{0}\left(t\right)\right]e^{\left(\beta_{1}X_{1}+\beta_{2}X_{2}+\beta_{3}X_{1}*X_{2}\right)}$$

where *h*(*t*) is the hazard function in time *t*, *h*_0_(*t*) corresponds to the basal hazard and *β* is the expected change in log-relative hazard for a unit of change in a shading level (*X*_*1*_), irrigation (*X*_*2*_), and the effect of the interaction between shading and irrigation (*X*_1_*X_*2*_).

## Results

### Seed germination

**Fig 1:**
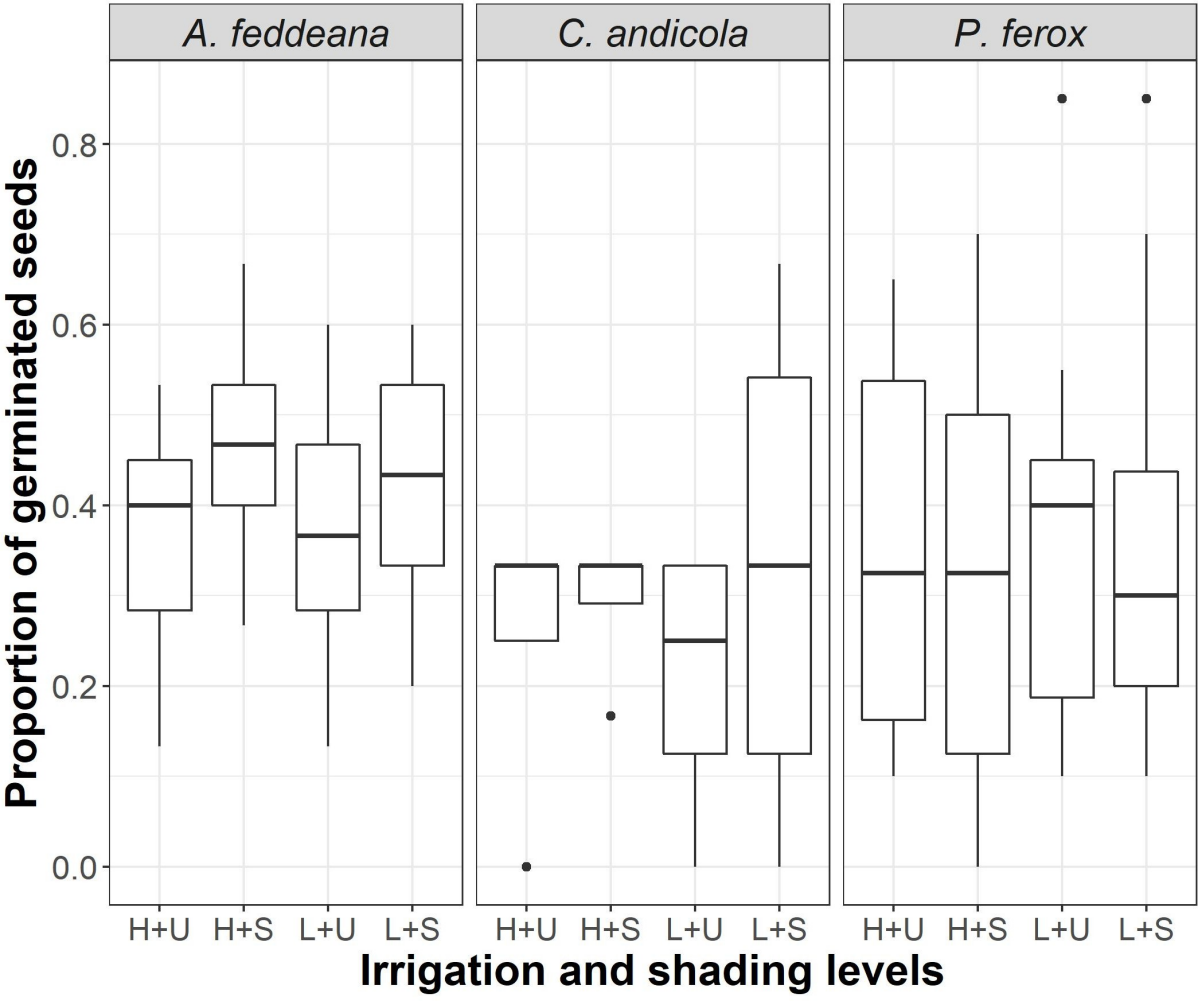
Effect of irrigation and shading levels on the germination of *Acacia feddeana*, *Cercidium andicola*, and *Prosopis ferox*. H: High irrigation (80 mm), L: Low irrigation (50 mm), U: Unshaded, S: Artificial shaded.

**Fig 2:**
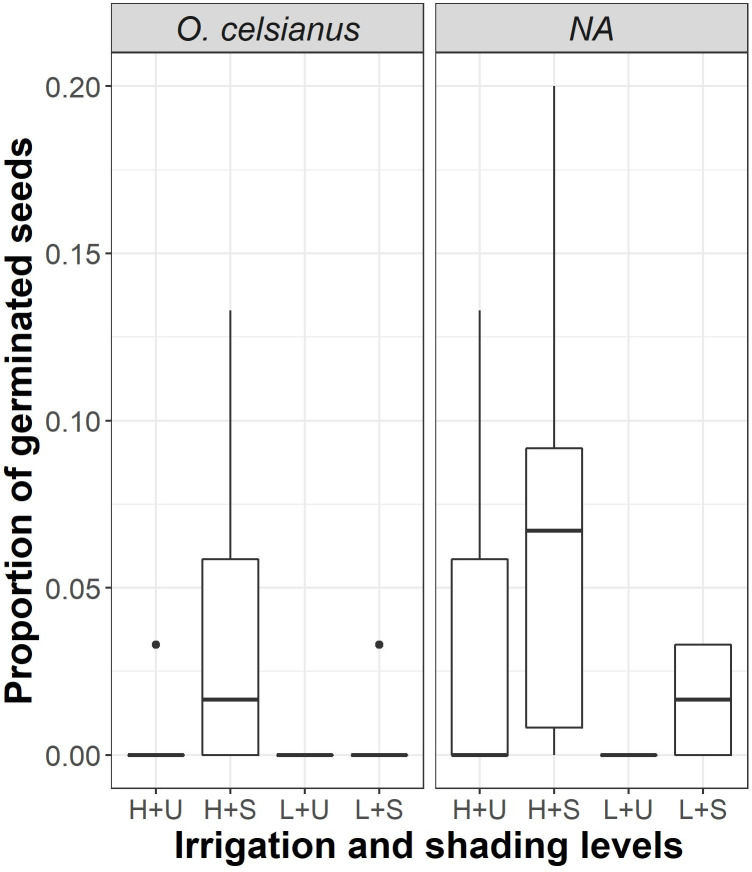
Effect of irrigation and shading on the germination of *Oreocereus celsianus* and *Parodia maassii*. H: High irrigation (80 mm), L: Low irrigation (50 mm), U: Unshaded, S: Artificial shaded.

The seeds of *Acacia feddeana* germinated between the 9th and 21st days, with a minimum germination threshold of ≈35 mm for all treatments. On the other hand, *Prosopis ferox* and *Cercidium andicola* seeds germinated with minimum irrigation of ≈40 mm between the 9th and 30th days. The seed germination of the studied species responded differently to the treatments, depending on the combination of shading and irrigation levels. The germination fraction (proportion) of three woody species, *Acacia feddeana*, *Cercidium andicola*, and *Prosopis ferox*, was not affected neither by shading nor irrigation level (Table 1, Fig 1, *A*. *feddeana—χ*^2^_(1)_ = 2.76, *P* = 0.097, *C*. *andicola*—*χ*^2^_(1)_ = 0.85, *P* = 0.36, *P*. *ferox—χ*^2^_(1)_ = 0.47, *P* = 0.49). In *Oreocereus celsianus*, both shading and irrigation levels affected the germination fraction (Table 1, Fig 2, *χ*^2^_(__2__)_ = 11.07, *P* = 0.0040). In *Parodia maassii*, shading level, irrigation level and their interaction affected germination percentage (Table 1, Fig 2, *χ*^2^_(__3__)_ = 29.19, *P* < 0.00001). In these two species, *O*. *celsianus* and *P*. *maassii*, germination reached its highest fraction with high irrigation (≈80 mm) and artificial shade. Neither species germinated in unshaded pots with low irrigation (≈50 mm), and *O*. *celsianus* had negligible germination in the other treatment combinations (Fig 2).

**Table 1:** The best model with at least one independent variable and null model of the generalized linear models of the germination percentage for each species.

Species	Error distribution	Models	*K*	Log L	AICc	ΔAICc	*P*
*Acacia*	Binomial	~ Shade	2	-81.5	167.3		0.097
*feddeana*		~ 1 (null)	1	-82.9	167.8	0.54	
*Cercidium*	Binomial	~ Shade	2	-22.9	50.7		0.358
*andicola*		~ 1 (null)	1	-23.3	48.9	-1.79	
*Prosopis*	Beta	~ Irrig	3	-112.3	231.3		0.494
*Ferox*	Binomial	~ 1 (null)	2	-112.6	229.4	-1.88	
*Oreocereus*	Beta	~ Irrig + Shade	4	-24.0	57.2		0.0040
*celsianus*	Binomial	~ 1 (null)	2	-29.6	63.4	6.25	
*Parodia*	Binomial	~ Irrig * Shade	4	-43.5	96.2		< 0.0001
*maassii*		~ 1 (null)	1	-58.1	118.4	22.15	

Irrig: irrigation levels, Shade: shading levels, *K*: the number of parameters in the model, ΔAICc: AICc min—AICc null, *P*: the *P*-value for the likelihood-ratio test.

### Seedling survival

**Fig 3:**
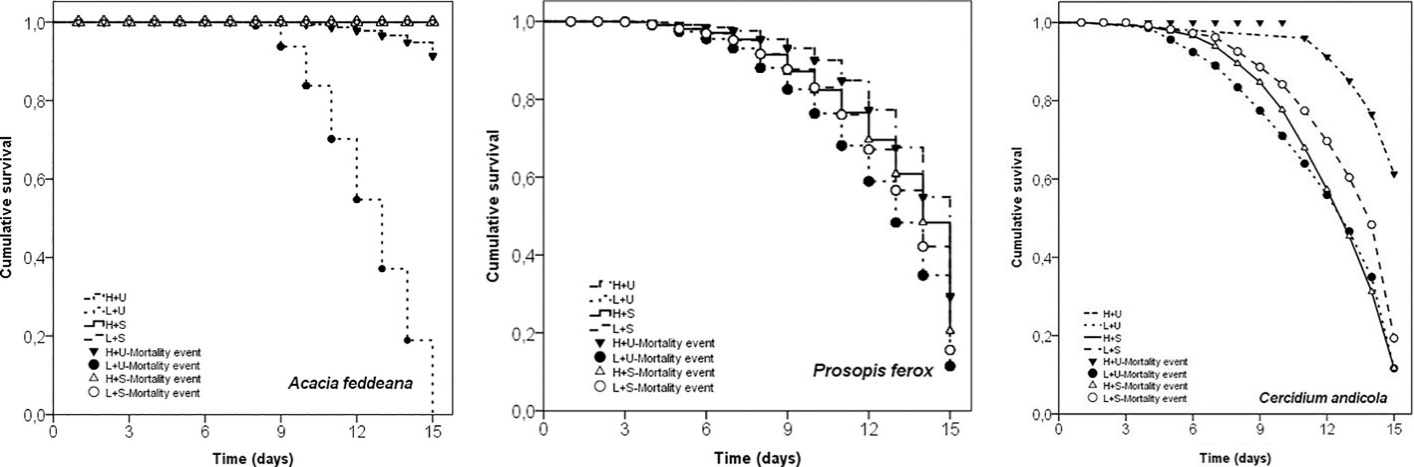
Comparison of survival curves among treatments by the Kaplan-Meier method for (a) *Acacia feddeana*, (b) *Prosopis ferox* and (c) *Cercidium andicola*. The lines show the treatments employed in the study. The symbol in each treatment indicates a mortality event. H: High Irrigation (80 mm), L: Low irrigation (50 mm), U: Unshaded, S: Artificial shaded.

Shading, irrigation, and their interaction affected the survival of *A*. *feddeana* seedlings (Cox model: Wald test[*X*^*2*^] = 4244; *P* < 0.001; Table 2). 100% of *A*. *feddeana* seedlings survived after 15 days in shaded pots, higher than unshaded pots. Especially, the seedlings in unshaded pots with low irrigation showed high and accelerated seedling mortality (Table 2; Fig 3A). There were also significant differences between the survival curves with a positive effect of shade and the high irrigation in the survival of *A*. *feddeana* seedlings (Kaplan-Meier method: Log Rank[*X*^*2*^] = 1120; *P* < 0.001; Fig 3A).

**Table 2:** Results of the Cox proportional hazards model for seedling survival.

Species	Variables	*(β)*	*Exp(β)*	*SE(β)*	*Z*	*P*
*A*. *feddeana*	Shaded	-17.360	2.901^−8^	1492	-16.245	<0.001
	Irrig 50mm	3.727	41.550	0.303	3.455	<0.001
	Shaded*Irrig 50mm	-3.727	0.024	2168	-3.572	<0.001
*P*. *ferox*	Shaded	0.661	1.937	0.076	4.647	<0.001
	Irrig 50mm	0.312	1.367	0.084	0.749	0.454
	Shaded*Irrig 50mm	-0.548	0.578	0.108	-1.088	0.277
*C*. *andicola*	Shaded	1.697	5.459	0.475	1.875	0.061
	Irrig 50mm	1.759	5.806	0.481	1.982	<0.05
	Shaded*Irrig 50mm	-2.139	0.118	0.534	-1.354	0.176

The negative coefficient (*β*) indicates that the factor has a positive effect on survival. In this case, the explanatory variables are shading levels (unshaded vs artificial shaded), irrigation (50 vs 80 mm) and their interaction.

Shade had negative effects on the survival of *P*. *ferox* seedlings (Cox model: Wald test[*X*^*2*^] = 25.14; *P* < 0.001; *1-exp β*), where the seedling mortality in shaded pots increased to 93,7% (Table 2, Fig 3B, Kaplan-Meier method: Log Rank[*X*^*2*^] = 82.78; *P* < 0.001). Seedling survival of *C*. *andicola* was not explained by shading and irrigation in the Cox model (Wald test[*X*^*2*^] = 4.14; *P* = 0.20); however, the seedlings in unshaded pots with high irrigation showed higher survival curves in the Kaplan-Meier method (Log Rank[*X*^*2*^] = 20.01; *P* < 0.001; Fig 3C).

## Discussion

The results of this study show that shade conditions and water availability, which is related to precipitation, affect the survival and establishment of plant species found in the Bolivian Prepuna. Higher precipitation and shade are important for the germination of cactus species and seedling survival of woody species, but not for the germination of woody species. In addition, the woody species responded differently to the shade condition: one species improved seedling survival under shade and the other two species showed higher seedling survival without shade.

Germination is a phenological, temporary event stimulated by climatic or environmental triggers. Noy-Meir [68], Schwining and Sala [69] indicate that the seeds of desert plants usually do not germinate after small rains, because they may be followed by drought. However, the rapid seed germination of species in arid regions such as those included in this study allows the species to take advantage of sporadic rains [70]. Thus, our results suggest that the minimum rainfall that triggers germination found for *Acacia feddeana*, *Prosopis ferox*, and *Cercidium andicola* are consistent, although higher, with the minimum rainfall found for other desert species of the genera *Cercidium* (19 mm) and *Encelia* (25 mm), among others [71, 72]. The germination threshold of these woody plants coincides with ca. 40 mm, reported by Lopez for Prepuna plants [52] and Ortega-Baes et al. [55].

The seeds of *A*. *feddeana*, *P*. *ferox*, and *C*. *andicola* germinated in similar proportions without exceeding 50% during the experiment, and the germination was not affected by soil water or shade availability. Therefore, it seems that relatively high-water availability does not foster seed germination of these woody species exposed to daylight, suggesting a typical response of a seed bank with innate dormancy [73], where similar fractions are always produced from year to year regardless of rainfall. However, the seeds of *P*. *maassii* and *O*. *celsianus* showed a positive response to water and shade, the combination of which probably promotes lower temperature stress and lower evaporation. Although shade allowed some germination of *P*. *maassii* even with low precipitation, *O*. *celsianus* was more restricted and only responded to high precipitation under shade. In any case, both species’ responses were predictive [74], which was also reported for annual plants of the Prepuna [59]. Thus, as for germination, our prediction was supported only for *P*. *maassii*.

Seedling survival of *A*. *feddeana* was high even without shade as long as there was abundant precipitation. However, the results suggest that in years with low rainfall, shade allows high survival in *A*. *feddeana*. Shade could become more important for this and other species in the future as the region is expected to become more arid [47, 48]. Shaded conditions imply lower temperatures as was recorded in our study in shaded pots, and this may allow establishment of drought-sensitive species, as lower temperatures are associated with higher relative humidity and less desiccating conditions. Other *Acacia* species also show similar high-water requirements. For example, *A*. *karroo* studied in Zimbabwe and Serengeti [75], and *A*. *tortilis* and *A*. *victorae* studied in a dry tropical forest [76], require high rainfall for recruitment and survival. On the other hand, *P*. *ferox* and *C*. *andicola* showed a more heliophilous behavior in terms of survival. In order to establish and survive, *P*. *ferox* and *C*. *andicola* seedlings need to be exposed to more sunlight, as long as there is high water availability. Seedlings of *Prosopis chilensis* also showed increased survival in open spaces compared with beneath nurse species [77]. In an observational study conducted by López et al. (2007), *P*. *ferox* seedlings seemed indifferent to shade, which could imply that different approaches (experimental/observational) may produce different results [78]. Therefore, in terms of survival, our general hypothesis was supported only for *A*. *feddeana* because each of the studied species showed a particular response to shade condition and irrigation.

Although survival of *O*. *celsianus* and *P*. *maassii* seedlings could not be determined, studies on cacti carried out for *Neobuxbaumia tetetzo* [9], *N*. *macrocephala* [79], *Mammillaria huitzilopochtli* [80], *N*. *macrocephala*, *N*. *mezcalaensis* [81] and *Carnegiea gigantea* [82] have shown that cactus survival depends on reductions of solar radiation and temperature, as well as increased soil moisture. The results for *P*. *maassii* and *O*. *celsianus* show that high water availability and shade are required to obtain at least low germination percentages; hence, shade would be an important factor for these species. The reduced temperatures under shade that likely were responsible for *A*. *feddeana* higher survival may have favored higher germination fractions in the cactus species, which are known to be sensitive to high temperatures [83]. Our results support that globose, opuntioid (not observed in this study), columnar cacti and *A*. *feddeana* more frequently establish spatial association with shrubs, due, most likely, to facilitation, in contrast to *Prosopis*, and *Cercidium* trees [28, 32, 84].

The shade projected by the canopy of trees or shrubs acts as a facilitating mechanism by reducing radiation and temperature [9, 85]. For the cactus *O*. *celsianus* and *P*. *maassii*, the effects of shaded pots on seed germination were positive, as they were on the survival of *A*. *feddeana* seedlings. Soil temperature reduction and lower evaporation prevail in shaded microsites, being the main factors that improve seed germination [15, 86]. The results obtained in this study corroborate this assertion, as the soil temperature within shaded pots was is in the range of 23–26°C versus 32–40°C in unshaded pots. These values agree with the optimal germination temperature of cacti, ca. 25°C [87]. These temperatures significantly favor *A*. *feddeana* seeds, coupled with an adequate soil moisture for germination and seedling survival found in shaded environments. On the other hand, the sheer nets played an important role in the reduction of radiation, which was 472.6 ± 55.8 Cd/m^2^, compared to the microsites without shade, where the incidence of light was 741.7 ± 176.7 Cd/m^2^. Therefore, the high humidity in the soil allowed 100% of *A*. *feddeana* seedlings to survive until the end of the experiment. In summary, plant water demand was reduced by shade when there was low water availability, e.g. Holmgren [30], Prider and Facelli [25].

How can we extend these results to the natural conditions, where environmental variability provides much more complexity to the species-environment relationship? The five study species coexist in many Prepuna localities, where the woody species tend to be dominant and compete [88]. We suspect that the coexistence was achieved partially by having different microhabitat preferences. We have documented that they differ at least in survival requirements. If we classify them from less to more heliophilous, we have *O*. *celsianus*, *P*. *maassii*, *A*. *feddeana*, and *C*. *andicola*≈*P*. *ferox*, in that order. The shade provided by small trees may facilitate the establishment of *A*. *feddeana* and cactus species (e.g. Valiente-Banuet and Ezcurra [9]; Rojas-Aréchiga et al. [89]; Rojas-Aréchiga and Vázquez-Yanes [87]). The positive association between *P*. *ferox* and *P*. *maassii* has been reported twice [32, 84]. In one case, a very strong spatial association was detected between this cactus species and medium sized individuals of *P*. *ferox* (individuals of around 50–100 cm) [78]. These individuals create relatively mild shaded conditions, suggesting that deep shade may not benefit this species. Different studies have addressed the role of the degree of shade [30, 90, 91], and they have found indeed that deep shade may even be detrimental for establishment [30, 92, 93]. How different levels of shade affect seedling response will have to be the subject of interest of future papers.

Species of the genus *Prosopis* are known for their intolerance to shade [94–96]. This information coincides with our results obtained for *P*. *ferox* which showed high mortality (94%) in shaded pots, indicating that this species is also shade-intolerant (heliophilic). There are many examples of cactus and woody seedlings distributed beneath shrub canopies, such as *Parodia maassii* [84], *P*. *flexuosa* and *Dodonaea viscosa* [97], and herbaceous seedlings under shrub canopies at the patch, community, and regional levels in the Prepuna biogeographical region [33, 53]. In our study, shade improved the germination percentage of *P*. *maassii*, *O*. *celsianus* and prolonged the survival of seedlings, especially of *A*. *feddeana*. In contrast, for *P*. *ferox* and *C*. *andicola* the effect of shade and precipitation had a neutral or even negative effect. This is another example of differences in the regeneration niche, which may contribute to species coexistence in the Prepuna. In addition to the positive effects of shade, edaphic factors can also play a role [26, 98, 99].

This study is one of the first to show key autecological aspects of the dominant species of one of the least known ecoregions of Argentina and Bolivia, the Prepuna, and has thus contributed to understanding facets of the dynamics of the arid ecosystems of the tropical and subtropical Andes in general, which share the characteristic of having rains almost strictly restricted to summer. We consider that this study, by incorporating the dominant species of the Prepuna, constitutes a contribution not only in terms of the knowledge of the ecology of plant species and the factors that affect them, but also of the conservation of other arid zones of South America.

## Supporting information

S1 Data An excel file containing the abiotic data. (XLSX)

S2 Data An excel file containing the germination and survival data. (XLSX)
